# Antioxidant and Anticlastogenic Capacity of Prickly Pear Juice

**DOI:** 10.3390/nu5104145

**Published:** 2013-10-18

**Authors:** Eduardo Madrigal-Santillán, Fernando García-Melo, José A. Morales-González, Patricia Vázquez-Alvarado, Sergio Muñoz-Juárez, Clara Zuñiga-Pérez, Maria Teresa Sumaya-Martínez, Eduardo Madrigal-Bujaidar, Alejandra Hernández-Ceruelos

**Affiliations:** 1 Laboratorio Medicina de Conservación, Escuela Superior de Medicina, IPN, Plan de San Luis y Díaz Mirón s/n, Unidad Casco de Santo Tomas, México D.F. 11340, Mexico; E-Mails: eomsmx@yahoo.com.mx (E.M.-S.); jmorales101@yahoo.com.mx (J.A.M.-G.); 2 Instituto de Ciencias de la Salud, Universidad Autónoma del Estado de Hidalgo, Ex-Hacienda de la Concepción, Pachuca, Hidalgo 42080, Mexico; E-Mails: electronicfer@hotmail.com (F.G.-M.); condesadetoledo@hotmail.com (P.V.-A.); sergiomzjz@gmail.com (S.M.-J.); zupecl@yahoo.com.mx (C.Z.-P.); 3 Secretaria de Investigación y Estudios de Posgrado, Universidad Autónoma de Nayarit, “Ciudad de la Cultura Amado Nervo”, Boulevard Tepic-Xalisco S/N. Tepic, Nayarit 28000, Mexico; E-Mail: teresumaya@hotmail.com; 4 Laboratorio de Genética, Escuela Nacional de Ciencias Biológicas, IPN, Av. Wilfrido Massieu, Unidad A. López Mateos, Zacatenco, Mexico D.F. 07700, Mexico; E-Mail: eduardo.madrigal@lycos.com

**Keywords:** prickly pears, anticlastogenic capacity, micronucleus assay, methyl methanesulfonate

## Abstract

Plants belonging to the genus *Opuntia* spp. are the most abundant of the Cactaceae family, grown throughout America and the Mediterranean central area. Its fruit, known as cactus pear or prickly pear, is an oval berry grouped in different colors. Some studies have shown its antioxidant activities which may help in preventing chronic pathologies such as diabetes. The purpose of the study was to evaluate the antioxidant capacity of three varieties of prickly pear juice (red-purple, white-green and yellow-orange) in five different concentrations (100, 250, 500, 750, and 1000 mg/mL) by DPPH (1,1-diphenyl-2-picrylhydrazyl radical) colorimetric method, selecting the best variety to determine its anticlastogenic potential against methyl methanesulfonate (MMS). The results indicate that the highest antioxidant was found in the juice of the prickly pear red-purple variety (PPRP), in all concentrations. Its anticlastogenic potential was therefore evaluated with a micronucleus assay. The experiment was run over two weeks. A negative control was included along with a positive control with MMS (40 mg/kg), a group of mice treated with PPRP (25 mL/kg), and three groups with PPRP (in doses of 25, 16.5 and 8.3 mL/kg) plus the mutagen. The PPRP was administered daily by oral gavage and the MMS was injected intraperitoneally five days prior to the end of the experiment. Blood samples were obtained at 0, 24, 48, 72 and 96 h in order to determine the frequency of micronucleated polychromatic erythrocytes (MNPE). The results indicated that PPRP is not a genotoxic agent, on the contrary, it may reduce the number of MNPE. In this regard, the PPRP showed an anticlastogenic effect directly proportional to its concentrations. Thus, the highest protection was obtained with a concentration of 25 mL/kg after 48 h of treatment.

## 1. Introduction

Plants belonging to the genus *Opuntia* spp*.* are the most abundant of the Cactaceae family, grown throughout the Americas as well as the central area of the Mediterranean, Europe, Asia, Africa, and Australia. *Opuntia* species display flattened stems called “pencas” or cladodes [1]. The cactus pear fruit also called prickly pear fruit is an oval elongated berry, with a thick pericarp, a juicy pulp with a considerable number of seeds and a semi-hard rind with thorns. The pericarp and the edible pulp may have different colors such as green, greenish white, canary yellow, lemon yellow, red, cherry-red, or purple hues [2]. The average weight of prickly pears fruits varies from 100 to 160 g depending on the origin site and cultivation. The usable part of the fruit is composed of peel (48%–52%) and pulp (48%–52%). The pulp can be further subdivided into seeds and strained pulp (44%–45%), the latter being the basis for fruit and juice products. The fruits with white pulp and green rind are preferred for consumption as food, and their domestic production corresponds to almost 95% of the total production [2]. Mexico is the main producer of *Opuntia ficus-indica* (L.) Mill species, and accounts for more than 45% of the worldwide production; however, only 1.5% of this production is exported [3].

Prickly pear fruit has long been known in traditional medicine for treating a number of pathologies such as ulcer, dyspnea, and glaucoma, as well as liver conditions, wounds and fatigue [4,5]. Different studies using European and Asian varieties of cactus pears have shown notable antioxidant activities that reduce significantly the oxidative stress in patients and may prevent chronic pathologies. In this sense, some preparations of fleshy stems (cladodes) have been tested for the treatment of diabetes symptomatology in humans and animal models [5,6,7]. Some authors have also reported that the fresh stems and nopal are a good source of fiber that also helps to reduce the blood sugar and plasma cholesterol levels [8,9]. The cactus pear fruit may be considered a functional food; this feature has been attributed to its bioactive compounds such as vitamin C and vitamin E, polyphenols, carotenoids, flavonoid compounds (e.g., kaempferol, quercetin, and isorhamnetin), taurine and pigments [10,11,12]. Betalains are water-soluble pigments. Two betalain derivatives are present in cactus-pears: betacyanin, responsible for their red-purple color, and betaxanthin, for their yellow-orange color. These pigments have shown beneficial effects on the redox-regulated pathways involved in cell growth and inflammation and have not shown toxic effects in humans [13,14]. Although the cactus pear fruit has shown several beneficial effects, there are few reports in the literature that demonstrate its genotoxic and/or antigenotoxic potential. Siriwardhana *et al.* demonstrated through the comet assay that a cactus pear extract in a 0.1 mg/mL concentration may reduce the H_2_O_2_-induced DNA damage in human peripheral lymphocytes [15]. Another study showed the ability of cactus cladodes to protect Balb/c mice against the genotoxicity of zearalenone (ZEN) with an efficient prevention of micronuclei, chromosomal aberrations frequency in bone marrow cells and DNA fragmentation, in comparison to the group treated with ZEN alone [16]. The purpose of the present study is to evaluate the antioxidant capacity of three varieties of prickly pear juice (red-purple, white-green and yellow-orange) in four different concentrations by DPPH (1,1-diphenyl-2-picrylhydrazyl radical) colorimetric method, and to select the best variety to determine its anticlastogenic potential against methyl methanesulfonate (MMS).

## 2. Experimental Section

### 2.1. Chemicals

The following compounds were purchased from Sigma Chemicals (St. Louis, MO, USA): diphenylpicrylhydrazyl, methyl methanesulfonate, α-tocopherol (vitamin E), and ethanol. The Giemsa stain was obtained from Merck (Mexico City, Mexico). Methanol, monobasic potassium phosphate (KH_2_PO_4_) and sodium phosphate dibasic (Na_2_HPO_4_) were acquired from JT Baker (Mexico City, Mexico).

### 2.2. Animals

All the animals were male mice strain NIH with a mean weight of 30 g. They were obtained from the Institute of Health Sciences-UAEH (Pachuca, Hidalgo, Mexico), and maintained under standard conditions in metallic cages at a temperature of 23 ± 2 °C and 50% ± 10% humidity, with food (TEKLAD Global 2018S, Harlan, Mexico City, Mexico) and water *ad libitum*, in a 12 h light-dark period. The adjustment time period for the animals was one week before the treatments.

### 2.3. Experimental Determinations

#### 2.3.1. Obtaining and Preparation of Three Varieties of Prickly Pear Juice

The three varieties of prickly pear fruit (red-purple, white-green and yellow-orange) used in the experiment were obtained from crops grown in the community of Emiliano Zapata, a town located in the State of Hildalgo, Mexico. The best pieces were selected from the three varieties and cleaned with tap water.

The three prickly pear juices were extracted with a juicer machine (Juice Extractor Multi TU67 Turmix, Mexico City, Mexico); subsequently, the juices were strained and filtered with paper Whatman No. 1 under vacuum. Finally, they were stored frozen (−4.0 °C) until use.

In a previous study, was evaluated the content of total phenolic compounds, ascorbic acid, betacyanin, betaxanthin and the stability of betacyanin pigments in the presence of Cu (II)-dependent hydroxyl radicals (OH) of the three varieties of fruit [1].

#### 2.3.2. DPPH Radical Scavenging Activity

The method used was the one described by Burtis and Bucar (2000) [17]. Methanolic solutions of the three varieties of prickly pear (100, 250, 500, 750, and 1000 mg/mL), and α-tocopherol (1 mg/mL) were prepared. Then, aliquots of 50 µL of each solution were added to 5 mL of a methanolic solution of DPPH (0.04%) and vortexed. Every assay was repeated 5 times. The absorbance was measured at 517 nm (Spectrophotometer 6405 UV/Vis Jenway, Cielo Vista, CA, USA), 30 min after the addition of the tested substances to the colored reactive. The concentrations tested were calculated according to the density of each juice (red-purple (1.023), white-green (1.033), and yellow-orange (1.016)), and were expressed in mg/mL in order to be compared with the α-tocopherol (Vitamin E).

#### 2.3.3. Genotoxicity/Antigenotoxicity Protocols

The protocol was approved by the Committee of Ethics and Biosecurity of the Institute of Health Sciences. The animals were organized each in groups of six individuals, as follows: a negative control group (without any treatment, only distilled water), a positive group treated with MMS (40 mg/kg body weight), a group administered with 25 mL/kg of the prickly pear variety red-purple (PPRP), and three groups treated with methyl methanesulfonate and the PPRP juice (in doses of 25, 16.5 and 8.3 mL/kg bw) respectively. The PPRP was chosen for having the highest antioxidant capacity and was administered daily by intragastric gavage for a two-week period and the MMS was injected intraperitoneally 5 days before the end of the experiment (single dose). The doses were selected considering a daily consumption of prickly pear juice of a person weighing 70 kg and correspond to: 8.3 mL/kg to 600 mL, 16.6 mL/kg to 1200 mL, and 25 mL/kg to 1750 mL.

The micronucleus assay was used to determine the genotoxic and antigenotoxic capacities of the compounds. At the end of the two-week treatment, blood samples were collected five times (at 0, 24, 48, 72 and 96 h) to determine the frequency of micronucleated polychromatic erythrocytes (MNPE).

Two blood smears were made from the tail of each animal, fixed in methanol for 5 min and stained for 20 min with a 4% Giemsa solution made in phosphate-buffered saline, at a pH of 6.8 [18]. One thousand erythrocytes per animal were scored to determine the rate of MNPE. In order to evaluate the bone marrow cytotoxicity, we scored 1000 erythrocytes per animal and established the rate of polychromatic erythrocytes (PE) with respect to the number of normochromatic erythrocytes (NE) (PE/NE index).

#### 2.3.4. Statistical Analysis

Data obtained were analyzed using the statistical program GRAPHPAD INSTAT version 3.0 (San Diego, CA, USA) ANOVA to determine if the data were normally distributed within all groups. Thereafter a Student *t* test was performed to establish the statistical differences between groups in each assay.

## 3. Results

**Figure 1:**
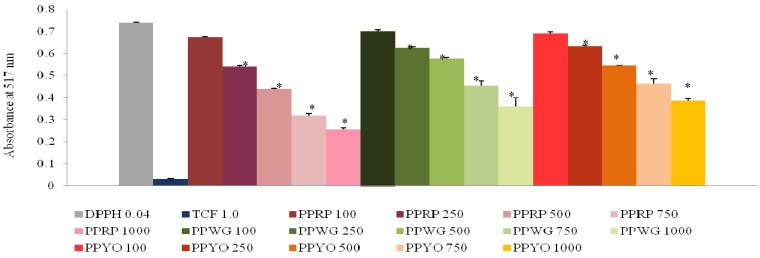
Total antioxidant activity of α-tocopherol (TCF) and three varieties of prickly pear fruit—red-purple (PPRP), white-green (PPWG), and yellow-orange (PPYO)—evaluated with the DPPH (1,1-diphenyl-2-picrylhydrazyl radical) colorimetric assay. * Statistically significant difference with respect to the value of the DPPH group. ANOVA and Student *t* tests, *p* ≤ 0.05.

Figure 1 shows the scavenging capacity of the three varieties of prickly pear fruit (red-purple, white-green and yellow-orange) determined with the DPPH method. The positive control (1 mg/mL of α-tocopherol) produced an inhibition of 90%. With respect to the prickly pear juice vials, we found that the three varieties of prickly pear showed a concentration dependent antioxidant capacity. However, the best inhibition corresponded to the red-purple juice variety, reaching an inhibition of 65% with the highest concentration (500 mg/mL).

**Figure 2:**
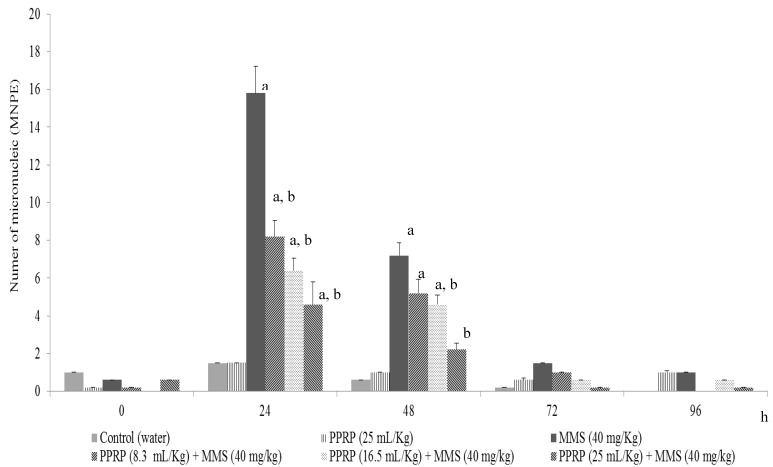
Frequency of micronucleated polychromatic erythrocytes (MNPE) in mice treated with juice of prickly pear variety red-purple (PPRP) and methyl methanesulfonate (MMS).Values represent the mean ± SD of six mice per group. The superscript letters show statistically significant differences as follows: ^a^ with respect to the control value; and ^b^ with respect to the value in the group treated with MMS. Anova and Student *t* tests (*p* ≤ 0.05).

The frequency of MNPE in the studied groups is shown in Figure 2. Animals belonging to the control and PPRP groups had no micronucleus increase during all experiments; the mean value was 0.8 MN/1000 PE. The result suggests that the PPRP is not genotoxic. On the other hand, the mice treated with methyl methanesulfonate (40 mg/kg) manifested a significant increase at 24 and 48 h of treatment, with the highest genotoxic damage of 15.8 MN/1000 PE at the 24 h. With respect to the animals administered with prickly pear red-purple variety before the MMS treatment, they showed a significant dose dependent reduction in the MNPE rate for all doses of PPRP tested. The highest protection (approximately, 70%) was obtained with 25 mL/kg of PPRP at 24 and 48 h.

The results of the relation between PE and the number of normochromatic erythrocytes (PE/NE index) showed the following: (a) At the beginning of the experiment, the PE/NE index was similar in all groups; at successive times, the control group as well as the animals treated with 25 mL/kg of prickly pear red-purple variety showed no significant variation in the index; (b) the mice administered with MMS showed a slight tendency to reduce the rate of PE at 72 h, however, this value was not statistically significant; and (c) all groups combined with PPRP and methyl methanesulfonate showed similar values in the PE/NE index; therefore, no significant differences were observed between them.

## 4. Discussion

In recent years there has been increasing attention paid to studying natural products that can counteract the damaging effects of environmental toxic compounds and/or prevent the development of many human diseases; thus, different types of fruits and vegetables have been evaluated and recognized as valuable sources of nutraceuticals. It is important to remember that this term incorporates the concepts “nutrition” and “pharmaceutical”, and is defined as a food (or part of a food) that provides health benefits, including prevention and/or treatment of chronic diseases [19]. Various studies have shown the functional properties of the prickly pear, which is why different researchers consider it a good natural source of nutraceuticals [2,20,21]. Among the phytochemicals found in its chemical composition to which the benefits in healthcare and disease prevention are attributed, belong vitamins, carotenoids, flavonoids and betalain pigments [12].

Nopal and cactus pears, as well as other fruits and vegetables, have been used in Mexican traditional medicine for the treatment of certain diseases such as ulcers, dyspnea and liver diseases [5]. Current evidence suggests that they have anti-inflammatory [22], anti-tumor [23], and antioxidant [24] capacities. The mechanisms exercising their health benefits are not fully established. Initially, studies addressed the presence of dietary fiber, but now, the nutraceutical benefits are presumed to be derived from their antioxidant properties [25].

In the present study, we evaluated the antioxidant capacity of three varieties of prickly pear juice (red-purple, white-green and yellow-orange) because these species are frequently consumed in our country. Likewise, we chose the juice while considering that most studies available in the literature are focused on the analysis of extracts of the prickly pear fruit or the nopal. Furthermore, in Mexico this fruit is usually consumed as a fresh fruit or juice, and to a lesser extent, as dried fruit, candies, jams, jellies and/or wines [26].

There are various methods to evaluate the antioxidant activity [27,28] either *in vitro* or *in vivo*. One of the strategies implemented in the *in vitro* tests on the antioxidant capacity of a compound or food, is to determine the activity against chromogenic substances of a radical nature in order to measure the loss of color in proportion to the concentration. However, the determinations of the antioxidant capacity performed *in vitro* only give us a rough idea of the events in complex situations *in vivo*, which has been a very useful tool to prove that the consumption of antioxidant substances can reduce oxidative stress in an organism [29]. In general, the antioxidant potential of a mixture or a fruit is determined not only by a substance, but it is the sum of the antioxidant capabilities of each of its components. Thus, the compounds present in the fruit can interact with each other to give synergistic effects [30,31]. The methods which are mostly applied are ABTS and DPPH. Both have excellent stability under certain conditions, although they also show certain differences [27,28]. In our study, we selected the DPPH method to evaluate the antioxidant capacity of the prickly pear fruit, and similar to most studies in the literature, we believe that this method has some advantages over the other one. DPPH is a stable free radical that can be obtained directly without prior preparation, while ABTS has to be generated after a reaction that can be chemical (manganese dioxide, potassium persulfate) [3,24,32,33], enzymatic (peroxidase, myoglobin) and/or electrochemical [31]. ABTS can measure the activity of compounds of both lipophilic and hydrophilic nature, while DPPH can only be dissolved in organic media, *i.e.*, polar solvents such as ethanol or methanol. Furthermore, the absorbance of the radical ABTS is at 414, 654, 754 and 815 nm in an alcoholic medium, while DPPH has an absorbance peak at 517 nm in aqueous solution [31,34]; which is a feature of the juice used in our experiment.

Our results showed that the three varieties of prickly pear juice have a potential antioxidant that depends on the concentration and that the best range capacity corresponded to the red-purple juice. Regarding the first point, we believe that this result is consistent because the antioxidant effect is attributed to polyphenols, which are the compounds that provide the color of the fruit. Thus, a significant increase in the consumption of prickly pear might also increase the antioxidant capacity. This hypothesis can be supported by the evidence that red berries (such as cranberry and blackberry) have a high antioxidant and chemopreventive potential [35], as well as on data from some other trials where we used the same kind of prickly pear (*O. ficus-indica*). For example, the research by Butera *et al.* [36] that evaluated the inhibition of oxidative lipid peroxidation of methanol in the extracts of three Sicilian cultivars of prickly pear (yellow, red and white) in red blood cells exposed to organic hydroperoxides. Their results showed that the extract from white and red fruits was the most effective in the inhibition of lipid oxidation, when compared to the α-tocopherol as a control reference, and further, that protection was dose-dependent [36]. In addition, studies conducted by Siriwardhana *et al.* who evaluated the antioxidant activity of an extract of cactus pear fruit (CPFE) against the effect of H_2_O_2_ in DNA showed that the reduction of damage to the genetic material in human peripheral lymphocytes was attributed to the CPFE constituents (mainly flavonoids and betalains) which were shown to be potent antioxidants *in vitro* with a concentration dependent activity [15].

With respect to the best antioxidant effect, our data suggest that the PPRP has a higher proportion of betanins, to which we can attribute the beneficial effects found. It is important to remember that the different colors of the fruit are caused by the combination of two kinds of pigments (purple-red betanin and the yellow indicaxanthin) and it has been observed that the amount of each pigment may vary with the type of fruit [5,32,36,37]. Two studies that analyzed the nutritional content and antioxidant capacity of several varieties of prickly pear, commonly grown and consumed in our country, indicated that both classes of pigments can be found in the fruits. Their results agree, in general, that indicaxanthin is present in light-colored fruits while betacyanins prevail in purple pigmented fruits which are those with the highest antioxidant activity. Besides, among the varieties tested in both studies, which showed the highest antioxidant capacity known, was moradillo (*O. violaceae*) and rastrero (*O. rastrera*) [32], as well as, pelota roja (*O. robusta*), and roja lisa (*O. ficus-indica*) [38]. Likewise, the authors believe that the antioxidant capacity may differ according to the method used; for example, when using the DPPH method, this property is attributed more to the presence of betalaines and vitamin C, which are compounds that are preferably analyzed in hydrophilic extracts. By contrast, using the FRAP method (ferric ion reducing antioxidant) the effect is directed to the presence of carotenoids, tocopherols and some phenolic compounds (such as flavonoids), which are identified more in the lipophilic extracts [32,38]. The combination of our research and the previous experiments suggests that if all these phytochemicals are present in the prickly pear, its antioxidant benefits may be attributed to a synergistic effect between betalains and flavonoids.

In the last 20 or 30 years, oxidative stress has been identified as the main component of several biological and pathological processes such as aging, inflammation, carcinogenesis, mutagenesis and diseases such as Parkinson’s and Huntington’s [24,39,40]. This has focused attention for a number of scientists to study the natural products that can counteract the damaging effects of oxidative stress and prevent multiple human diseases. This phenomenon has been called chemoprevention. In the case of the prickly pear, as already mentioned, several studies demonstrate its antioxidant functions, however, information on its chemopreventive capacity is not yet complete and the existing information is still widely dispersed. In the present study it has also been demonstrated that the juice of PPRP is not a genotoxic or cytotoxic agent, however, it may significantly reduce the clastogenicity produced by MMS. It is noteworthy that in the literature there are few *in vivo* studies that have used the same genotoxic and/or antigenotoxic indicator, with the same species of prickly pear. Researches by Zorgui *et al.* [16] demonstrated that the administration of an extract of cactus cladodes may protect Balb/c mice of six weeks of age against genotoxic damage induced by mycotoxin zearalenone (ZEN). Their results showed that the ethanol extract of the prickly pear in the highest dose (100 mg/kg) was completely safe and did not induce any genotoxic effect. Conversely, when administered concurrently with ZEN (at a dose of 40 mg/kg bw), we observed a significant decrease of micronuclei in the frequency of chromosome aberrations evaluated in bone marrow cells as well as DNA fragmentation when compared to the group treated only with ZEN [16]. On comparing this experiment with ours, we observe some matching data such as the cactus cladodes extract which is from the same species as the juice that we used (*O. ficus-indica*). The doses of the extract used by Zorgui were 25, 50 and 100 mg/kg and he found that the antigenotoxic protection was dose dependent, that is, an effect similar to the one we obtained. Although the doses we used were different and selected on the basis of the consumption of prickly pear juice per day by one person of 70 kg (25, 16.6 and 8.3 mL/kg), our results indicated that the best anticlastogenic effect corresponded to the highest dose, which suggests that by increasing the dose of juice, the effect will probably be similar. On the other hand, our experiment and the previous one showed that the cladodes cactus extract and PPRP were safe, by not inducing MNPE. So, we can state and justify the results of other authors when they say that the main phytochemicals (betalains and flavonoids) are also safe [5,41,42].

The main finding of this work has been to demonstrate the protection obtained by the juice from a variety of prickly pear fruit frequently consumed in México against damage caused by methyl methanesulfonate, which is an alkylating agent that has demonstrated its mutagenic, carcinogenic and teratogenic abilities in different trials [43]. In general, the toxic mechanism of action involves the direct alkylation by type 2 nucleophilic substitution of guanine and adenine, which results in their ability to induce single and double strand breaks, as well as to generate reactive oxygen species [33]. As a whole, the data suggest that the protective effect of the prickly pear juice red-purple variety is associated with the presence of phytochemicals of their own chemical composition, and although our data do not confirm it completely, we think that the anticlastogenic ability is probably attributed to a synergistic antioxidant mechanism between betalains and flavonoids as they are the main components. Considering that in the recent literature, some researchers have suggested that antioxidants compounds may perform other functions, independently of their ability to scavenge free radicals, and our results showed that the anticlastogenic effect was against a direct-acting alkylating agent with DNA; together this should promote the development of new investigations to explore the specific mechanisms of the antioxidant capacity of the prickly pear fruit—in particular, its direct interaction with nuclear receptors and its ability to modulate the activity of key enzymes involved in cell signaling and antioxidant responses [44]. The results of this study confirm that prickly pear juice can be considered an important chemopreventive agent to reduce the incidence of some diseases and complements the evidence of other authors who have used the prickly pear fruit against different toxic agents such as H_2_O_2_ [36], aflatoxin B_1_ [45], cisplatin [46] and benzo(a)pyrene [47]. Moreover, it has come to be considered a cancer chemopreventive agent [23].

## 5. Conclusions

In the present investigation we have demonstrated that treatment with PPRP prevents significantly damage induced by methyl methanesulfonate in mice, thereby reducing the frequency of micronuclei. Our data also suggest that the antioxidant capacity of the juice may be involved in the anticlastogenic effect.
